# Goose Nephritic Astrovirus Infection of Goslings Induces Lymphocyte Apoptosis, Reticular Fiber Destruction, and CD8 T-Cell Depletion in Spleen Tissue

**DOI:** 10.3390/v13061108

**Published:** 2021-06-09

**Authors:** Rui Ding, Han Huang, Hongyu Wang, Zewen Yi, Siyu Qiu, Yingjun Lv, Endong Bao

**Affiliations:** MOE Joint International Research Laboratory of Animal Health and Food Safety, College of Veterinary Medicine, Nanjing Agricultural University, Nanjing 210095, China; 2019107013@njau.edu.cn (R.D.); 2019807138@stu.njau.edu.cn (H.H.); wanghongyuwhy92@163.com (H.W.); 2020807153@stu.njau.esu.cn (Z.Y.); 2018107009@njau.edu.cn (S.Q.); b_endong@njau.edu.cn (E.B.)

**Keywords:** goose nephritic astrovirus, spleen, pathological changes, apoptosis, CD8

## Abstract

The emergence of a novel goose nephritic astrovirus (GNAstV) has caused economic losses to the Chinese goose industry. High viral load is found in the spleen of goslings infected with GNAstV, but pathological injuries to the spleen due to GNAstV are largely unknown. In this study, 50 two-day-old goslings were infected orally with GNAstV, and 50 goslings were treated with PBS as control. Spleens were collected at different times following infection to assess damage. GNAstV infection caused visceral gout and urate deposition in joints, and resulted in 16% mortality. GNAstV was found in the lymphocytes and macrophages within the spleen. Lymphocyte loss, especially around the white pulp, and destruction and decline in the number of reticular fibers was observed in GNAstV-infected goslings. Moreover, in GNAstV-infected goslings, ultrahistopathological examination found that splenic lymphocytes exhibited condensed chromatin and apoptotic bodies, and reticular cells displayed damage to plasma membrane integrity and swollen mitochondria. Furthermore, TUNEL staining confirmed apoptosis of lymphocytes, and the mRNA levels of Fas and FasL were significantly increased in the GNAstV-infected goslings. In addition, GNAstV infection reduced the number and protein expression of CD8. In conclusion, GNAstV infection causes lymphocyte depletion, reticular cell necrosis, reticular fiber destruction, lymphocyte apoptosis, and reduction in CD8 levels, which contribute to spleen injury.

## 1. Introduction

GNAstV is a novel pathogen that was first isolated in China in 2018 [1]. GNAstV is a small, non-enveloped, single-stranded RNA virus. GNAstV infection in three- to fifteen-day-old goslings causes visceral gout and results in 2–20% mortality. In addition, GNAstV infection has been observed in Cherry Valley ducklings in the clinic, and has also been shown to infect chickens in experimental studies [2,3,4]. At present, most studies have focused on the isolation and identification of GNAstV, animal regression, and establishing methods for detecting the virus [5,6,7]; however, little is known about the pathogenic mechanisms of GNAstV infection.

Goslings that are infected with GNAstV display swelling and urate deposition in kidney tissue, as well as liver damage. Both organs’ lesions are easy to understand because the liver is responsible for the production of uric acid, and the kidney is the main excretory organ. Additionally, a high viral load and necrosis of splenic lymphocytes was also observed in infected animals [8,9], indicating that GNAstV may cause damage to the spleen tissue and induce immunosuppression. Moreover, recent studies show that GNAstV infection increases pro-inflammatory cytokines, such as IL-1β and IL-8, and decreases MHC-II levels in the spleen at the early stages of infection [10,11]. In addition, a case of co-infection with the astrovirus and parvovirus in goslings was also reported [12], although it is not known whether GNAstV contributes to the transmission or spread of the goose parvovirus. Although there are sporadic reports of spleen damage in goslings infected with GNAstV, the specifics of these injuries need to be further investigated. In the present study, two-day-old goslings were experimentally infected with GNAstV, and changes in gross morphology, histopathology, ultrahistopathology, apoptosis, autophagy, and levels of CD4 and CD8 expression in spleen tissue were investigated.

## 2. Materials and Methods

### 2.1. Virues

The GNAstV-JSHA isolate (GenBank accession no. MK125058) was isolated and kept in our laboratory, and has been shown to cause visceral gout [13]. The titer of the virus was 1 × 10${}^{4.25}$ 50% tissue culture infective dose (TCID50)/mL, as determined by titration on goose kidney epithelial cells according to the method of Reed and Muench.

### 2.2. Animal Experiment

Before the animal experiment, five two-day-old goslings (Yangzhou white goose) were euthanized with intravenous pentobarbital sodium. No gross changes were found in all organs, and no GNAstV RNA was detected from kidneys, spleens and livers using the RT-PCR method. Then, 100 one-day-old goslings were purchased from the same flock and randomly assigned to two groups, namely, the infection group and control group, each with 50 goslings per group. These goslings were reared separately in negative-pressure animal isolators without any immunizations and provided with sterilized water and feed without antibiotics ad libitum. The goslings in the infection group were challenged with 0.5 mL (0.5 × 10${}^{4.25}$ TCID50/goose) GNAstV by oral infection. The control group was fed with 0.5 mL of PBS. All geese were monitored daily for the occurrence of clinical signs for 19 days. During the experiment, five geese from each group were weighed, blood-collected and euthanized with intravenous pentobarbital sodium at 12 h, 1, 2, 3, 5, 7, 11, 15 and 19 days post-infection (dpi). The spleen was collected and weighted, and the relative weights of the spleen were calculated as a proportion of each gosling’s body weight. Then, a portion of the spleen was fixed in 4% paraformaldehyde for histopathological examination. Part of the spleen was fixed in 2.5% glutaraldehyde for ultrahistopathological examination. The rest were stored at −80 ${}^{\circ }$C for further experiments.

### 2.3. Histopathological Examination

Hematoxylin and eosin staining was performed according to previous studies with minor revision [14]. Briefly, the spleen samples were fixed in 4% paraformaldehyde and dehydrated by a series of alcohols, clarified in xylene, and embedded in paraffin. Then, samples were sliced serially into 4 μm sections and stained with hematoxylin and eosin by routine methods. Stained sections were examined with a light microscope (Carl Zeiss, Gottingen, Germany).

### 2.4. Silver Staining

Silver staining was performed according to the instruction of a commercial Improved Godon and Sweet kit (Yifeixue Bio Tech, Nanjing, China). Briefly, after dewaxing to water, the spleens’ paraffin sections were oxidized by the Gordon and Sweet oxidant for 4 min, bleached by oxalic acid for 3 min, mordanted by ferric ammonium alum for 14 min, immersed by silver ammonia by 10 min, reduced by Gordon and Sweet reductant for 4 min, and counterstained by kernechtrot for 20 min.

### 2.5. Immunohistochemical (IHC) Examination

IHC staining was performed according to the conventional protocol with some modifications [15]. After deparaffinization and rehydration, the spleen tissue slides were inactivated with 3% hydrogen peroxide for 10 min, and then exposed to citric acid buffer at 100 ${}^{\circ }$C for 8 min. The sections were blocked with 5% bovine serum albumin for 30 min and incubated with mouse anti-GNAstV Capsid protein monoclonal antibody (gift from Prof. Zongyan Chen, Shanghai Veterinary Research Institute, China), rabbit anti-CD4 polyclonal antibody (Beyotime, Shanghai, China), and mouse anti-CD8 monoclonal antibody (gift from Prof. Bo Ma, Northeast Agricultural University, China) at 4 ${}^{\circ }$C overnight. After washing with PBS and incubation with secondary antibodies for 1 h at 37 ${}^{\circ }$C, the samples were visualized using diaminobenzidine staining and counterstained with hematoxylin. Stained sections were examined with a light microscope. The software Image-Pro Plus (Version 6.0.0.260) was used to conduct semi-quantitative statistics of the positive signals.

### 2.6. Ultrahistopathological Examination

The spleens were trimmed into 1 mm${}^{3}$ blocks, and then were dehydrated in ethyl alcohol, infiltrated with a propylene oxide-Araldite mixture, and embedded in Araldite. Then the ultrathin section was stained with uranyl acetate and lead citrate for 15 min. The samples were visualized under a HT7700 transmission electron microscope (Hitachi, Tokyo, Japan).

### 2.7. Virus Determination

Viral RNA was extracted from serum and spleen samples using the GeneJET Viral DNA/RNA Purification Kit (Thermo Scientific, Rockford, IL, USA) and transcribed into cDNA using the HiScript Q RT SuperMix Kit (Vazyme Biotech Co., Ltd., Nanjing, China). Then the viral loads were measured using a SYBR Green I-based real-time PCR method established in our lab [16].

### 2.8. Terminal Deoxynucleotidyl Tranferase-Mediated Nick-End Labeling (TUNEL) Staining

TUNEL staining was performed according to our previous study [17]. The tissue sections were deparaffinated in xylene and rehydrated in descending concentrations of ethanol, followed by antigen retrieval in sodium citrate buffer for 10 min at room temperature and in a microwave oven at 100 ${}^{\circ }$C for 15 min. Endogenous peroxidase was inhibited by incubation with 3% hydrogen peroxide for 15 min at room temperature. Then, tissue sections were stained by TUNEL using the ApopTag kit (Roche, Indianapolis, IN, USA) according to the manufacturer’s protocol. The sections were then stained with hematoxylin. The apoptotic index was calculated as the percentage of TUNEL-positive cells in spleen cells.

### 2.9. Quantitative Real-Time PCR (qPCR) Analysis

Total RNA was extracted using Trizol (Invitrogen, Carlsbad, CA, USA) and reverse transcription of the RNA was conducted using the HiScript Q RT SuperMix Kit (Vazyme Biotech Co., Ltd., Nanjing, China). A Thermocycler (Bio-Rad, Hercules, CA, USA) was used for quantitative PCR. Primer 5.0 software was used for the designation of the related genes, as shown in Table 1. RNA expression was normalized by quantification of GAPDH as a housekeeping gene. Each sample was prepared in three duplicate tubes. Specific gene expression was quantified using the 2${}^{-\Delta \Delta CT}$ method.

### 2.10. Western Blotting

The spleen samples were lysed in 500 μL RIPA lysis buffer supplemented with 1% Phenylmethanesulfonyl fluoride. Following centrifugation at 13,000× *g* for 15 min at 4 ${}^{\circ }$C, the concentration of the total protein was quantified using a BCA assay kit (Thermo Scientific, Rockford, IL, USA). The lysates were mixed with 5 × SDS loading buffer and heated at 99 ${}^{\circ }$C for 10 min. Samples were then analyzed by SDS-PAGE essentially, as previous described [19]. The rabbit monoclonal anti-GAPDH antibody (Abcam, Cambridgeshire, Britain), rabbit polyclonal anti-LC3B antibody (Abcam, Cambridgeshire, Britain), rabbit anti-Beclin1 polyclonal antibody (Beyotime, Shanghai, China), rabbit anti-CD4 polyclonal antibody (Beyotime, Shanghai, China) and mouse anti-CD8 monoclonal antibody were used as primary antibodies, and HRP donkey anti-mouse IgG or HRP donkey anti-rabbit IgG (ABclonal, Wuhan, China) were served as the secondary antibody in the study. Protein bands in the membrane were detected using a ChemiDoc Touch Imaging System (Bio-Rad, Hercules, CA, USA) after incubation of the membranes with Clarity Western ECL Blotting Substrate. Then, protein bands were analyzed by software Image-Pro Plus (Version 6.0.0.260), and protein levels were normalized to the amount of GAPDH.

### 2.11. Statistical Analysis

The differences between the control group and experimental group were analyzed by Student’s *t*-test. The results are expressed as the mean ± standard deviation (SD). *p* < 0.05 was considered to indicate a statistical significance compared with the control group, and *p* < 0.01 was considered to indicate a high degree of significance compared with the control group.

## 3. Results

### 3.1. Clinical Changes

**Figure 1:**
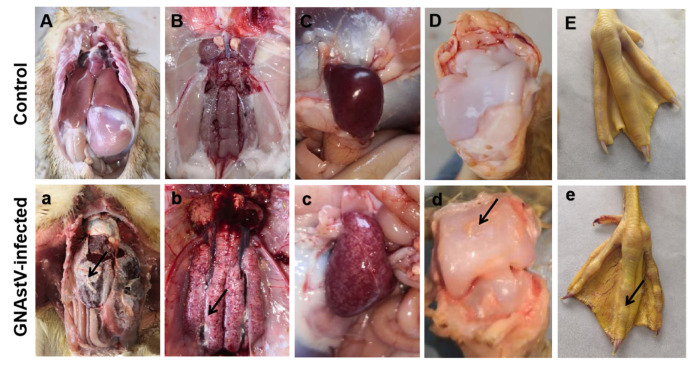
Gross anatomical changes in goslings associated with GNAstV-mediated mortality. No changes were observed in the liver (**A**), heart (**A**), kidney (**B**), spleen (**C**), articular cavity (**D**) and claw (**E**) in the control goslings. Urate deposition on the surface of the liver and heart (**a**), and in the ureter (**b**), articular cavity (**d**) and claw (**e**), swelling and pale coloration of the kidneys (**b**), and enlargement and necrosis of spleens (**c**) were observed in the infected goslings. Black arrow: urate.

The goslings were in good health and no dead ones were found in the control group. However, the goslings in the infected group showed a reduction in body weight from 5 dpi to the end of the experiment, and eight goslings died in total (1, 1, 2, 2, 1 and 1 gosling died at 3, 4, 5, 7, 8 and 10 dpi, respectively). The mortality was 16%. Urate disposition on the surface of the liver and heart and in the ureter and articular cavity, swelling and pale kidneys, and enlargement and necrosis of spleens at autopsy were observed in dead goslings (Figure 1a–e), while no obvious pathological changes were found in the control group (Figure 1A–E). For most surviving goslings, only pathological changes in the kidney and spleen were observed, while no obvious urate disposition was found.

### 3.2. Gross Morphological Changes in Spleen Tissue

**Figure 2:**
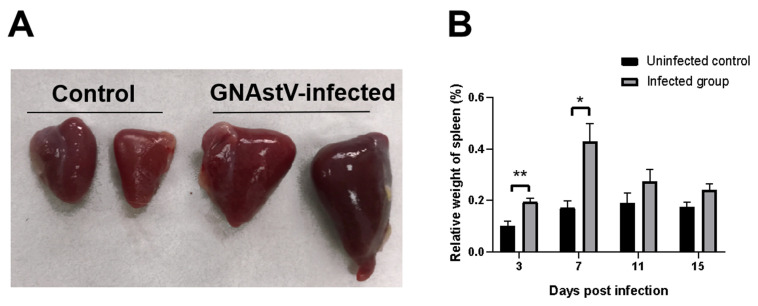
Infection with GNAstV induces gross morphological changes and increase in spleen weight in infected goslings. The spleens of the infected goslings were larger than in the control at 5 dpi (**A**). The relative weight of spleens was measured at 3, 7, 11, and 15 dpi (**B**). Values are expressed as mean ± SD, *n* = 5. * *p* < 0.05; ** *p* < 0.01.

Enlargement of the spleen and diffuse multifocal whitish necrotic spots in severely damaged spleens were observed in the infected group at 3 to 7 dpi compared to the control group (Figure 1c and Figure 2A). No obvious pathological changes were found at 11 and 15 dpi. The relative weight of the spleen at 3 and 7 dpi was significantly higher in the GNAstV-infected goslings than in the negative control group (Figure 2B).

### 3.3. Histopathological Changes in the Spleen

**Figure 3:**
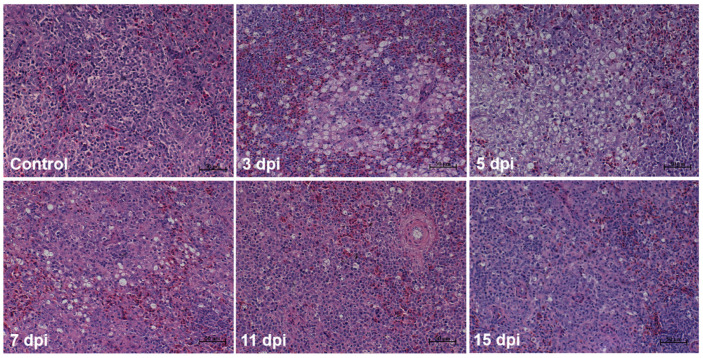
Histopathological changes in the spleen of goslings infected with GNAstV. Scale bar: 50 μm.

**Figure 4:**
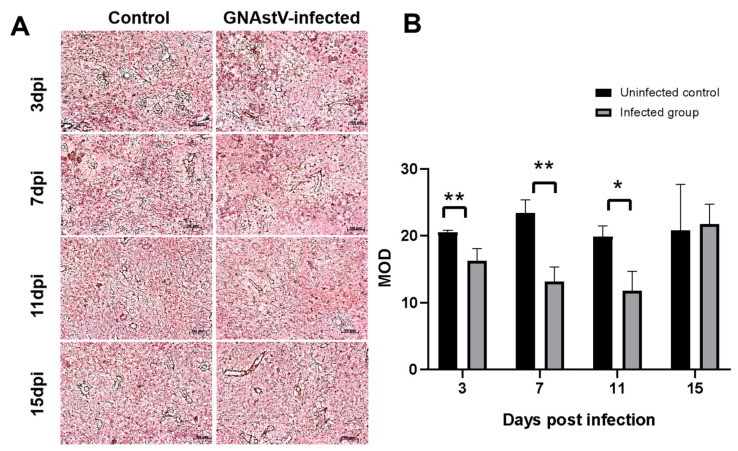
Changes in fibers associated with GNAstV infection. Spleen tissue was stained with the Gordon and Sweet method. The reticular fibers are indicated by black signals (**A**). The mean optical density (MOD) of reticular fibers was calculated by Image-Pro Plus (**B**). Scale bar: 50 μm. Values are expressed as mean ± SD, *n* = 5. * *p* < 0.05; ** *p* < 0.01.

On histopathological examination, the spleens from the control goslings appeared histologically normal. However, necrosis of splenic lymphocytes and a starry sky pattern were found in the spleens of the infected goslings, especially at the area between the white pulp and red pulp. Serious pathological damage was found at 3 and 7 dpi; this damage became alleviated after 7 dpi (Figure 3). Silver staining showed that the reticular fibers in the infected group were broken (Figure 4A), and the number of reticular fibers in the infected group was significantly lower than in the control at 3, 7, and 11 days (Figure 4B).

### 3.4. Virus Location and Viral Load in Spleen Tissue

**Figure 5:**
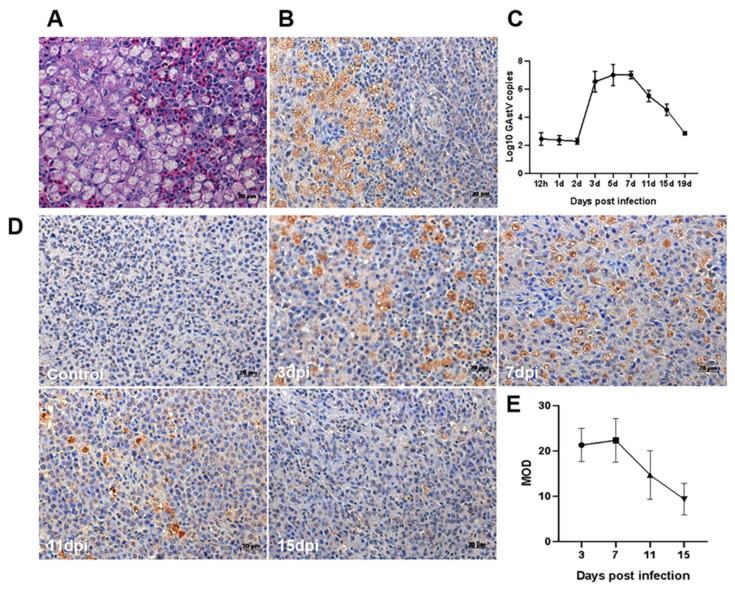
Virus location and viral load in the spleen. Spleen tissue was stained with hematoxylin and eosin (HE) (**A**), and the virus location was detected by IHC (**B**). Viral copy numbers in spleens in goslings infected with GNAstV were evaluated using real-time PCR (**C**). The virus location was detected by IHC (**D**), and the mean optical density (MOD) was calculated by Image-Pro Plus (**E**). Scale bar: 20 μm. Values are expressed as mean ± SD, *n* = 5.

Immunohistochemistry (IHC) was performed to detect the distribution of GNAstV in the spleen. GNAstV was observed in the cytoplasm of splenic lymphocytes and macrophages. Moreover, intense staining for the virus was found at the area between the white pulp and red pulp; this is consistent with the pathological damage observed in HE staining (Figure 5A). qPCR was performed to measure the viral load in the spleen, and the viral load was found to begin to increase at 2 dpi, and reached the peak at 7 dpi, then began to decrease (Figure 5B). The vial load at 19 dpi was almost the same as at the beginning of the infection (12 h). Similar results in viral load were observed in the IHC assays (Figure 5C,D).

### 3.5. Ultrahistopathological Changes in Spleen Tissue

**Figure 6:**
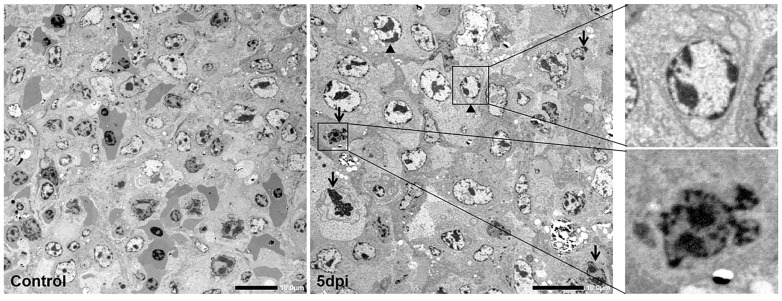
Ultrahistopathological changes in spleen induced by GNAstV infection. Arrow: Condensed chromatin and apoptotic cells among lymphocytes. Triangle: swollen mitochondria and impaired plasma membrane integrity in reticular cells. Scale bar: 10 μm.

The splenic lymphocytes and reticular cells from the control goslings are normal, and no obvious pathological changes were observed. However, some apoptotic cells were found among splenic lymphocytes of infected goslings, and these cells were characterized by sharply delineated masses of condensed chromatin and formation of apoptotic bodies (Figure 6, arrow). In addition, loss of plasma membrane integrity and swollen mitochondria were also observed in the reticular cells of infected goslings (Figure 6, triangle).

### 3.6. Effects of GNAstV Infection on Apoptosis of Splenic Lymphocytes

**Figure 7:**
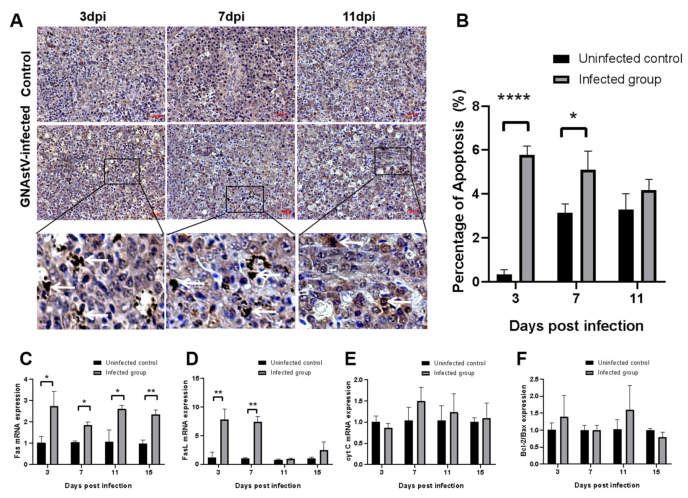
Effects of GNAstV infection on apoptosis of splenic cells. Paraffin-embedded sections of spleen tissue were stained by the TUNEL method (**A**). The apoptotic index was calculated as the percentage of TUNEL-positive cells in spleen cells (**B**). Changes in the mRNA levels of Fas (**C**), FasL (**D**), and cyt C (**E**), and the ratio of Bcl-2/Bax (**F**) in the spleen at 3, 7, 11, and 15 dpi in goslings infected with GNAstV were determined. White arrow: apoptosis of splenic cells. Scale bar: 20 μm. Values are expressed as mean ± SD, *n* = 5. * *p* < 0.05; ** *p* < 0.01; **** *p* < 0.0001.

TUNEL staining was used to evaluate apoptosis in splenic cells. As shown in Figure 7A, the positive signals (brown points) were observed in the splenic lymphocytes of control and infected goslings, and more positive signals were found in the infected goslings compared to the control. The percentage of apoptosis was significantly increased in spleens from the infected group at 3 and 7 dpi compared to the control group (*p* < 0.05), while there was no significant difference in apoptosis between the two groups at 11 dpi (Figure 7B; *p* > 0.05). The mRNA levels of Fas, FasL, cyt C, Bax, and Bcl-2 were determined by qPCR. Expression of Fas mRNA at 3, 7, 11, and 15 dpi and expression of FasL mRNA at 3 and 7 were higher in the infected group than in the control group (Figure 7C,D; *p* < 0.05). No differences were found in cyt C mRNA expression or in the ratio of Bax and Bcl-2 between the two groups (Figure 7E,F; *p* > 0.05).

### 3.7. Effects of GNAstV Infection on Autophagy of Splenic Cells

**Figure 8:**
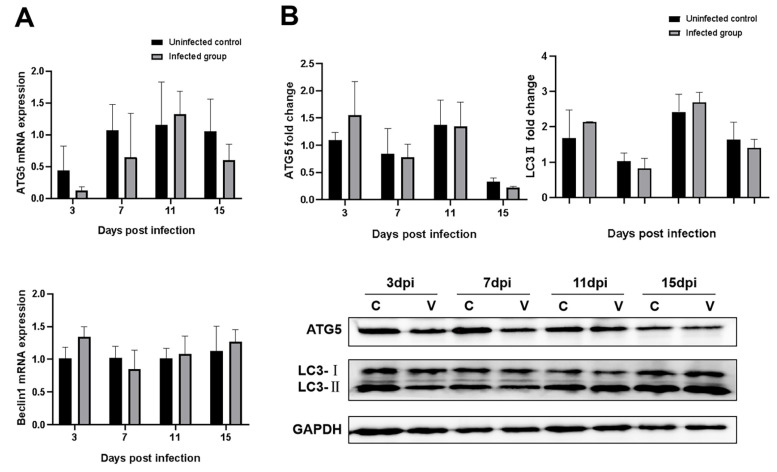
Effects of GNAstV infection on autophagy of splenic cells. Changes in the mRNA levels of ATG5 and Beclin1 in spleen tissue at 3, 7, 11, and 15 dpi after infection with GNAstV (**A**). Changes in the protein levels of ATG5 and LC3-II in spleen tissue at 3, 7, 11, and 15 dpi after infection with GNAstV (**B**). C: uninfected control group; V: GNAstV-infected group. Values are expressed as mean ± SD, *n* = 5.

To determine whether GNAstV infection influenced autophagy in the spleen, the mRNA expression of autophagy-related genes, including ATG5 and Beclin1, and levels of proteins including ATG5 and LC3II were measured by qPCR and Western blot, respectively. No differences in ATG5 and Beclin1 mRNA levels in the spleen were observed between the infected and negative control groups (Figure 8A, *p* > 0.05). There were no differences found in ATG5 and LC3II protein levels between the two groups (Figure 8B, *p* > 0.05).

### 3.8. Effects of GNAstV Infection on CD4 and CD8 Expression in the Spleen

**Figure 9:**
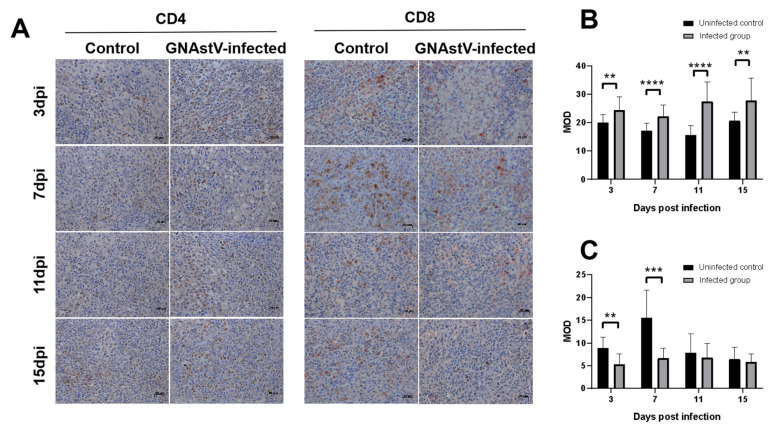
Immunohistochemical examination of the spleen using CD4 and CD8 antibodies. Changes in positive signals of CD4 and CD8 in the spleen at 3, 7, 11, and 15 dpi after infection with GNAstV (**A**). The mean optical density (MOD) of a CD4 positive signal (**B**) and CD8 positive signal (**C**) was calculated by Image-Pro Plus. Scale bar: 20 μm. Values are expressed as mean ± SD, *n* = 5. ** *p* < 0.01; *** *p* < 0.001; **** *p* < 0.0001.

**Figure 10:**
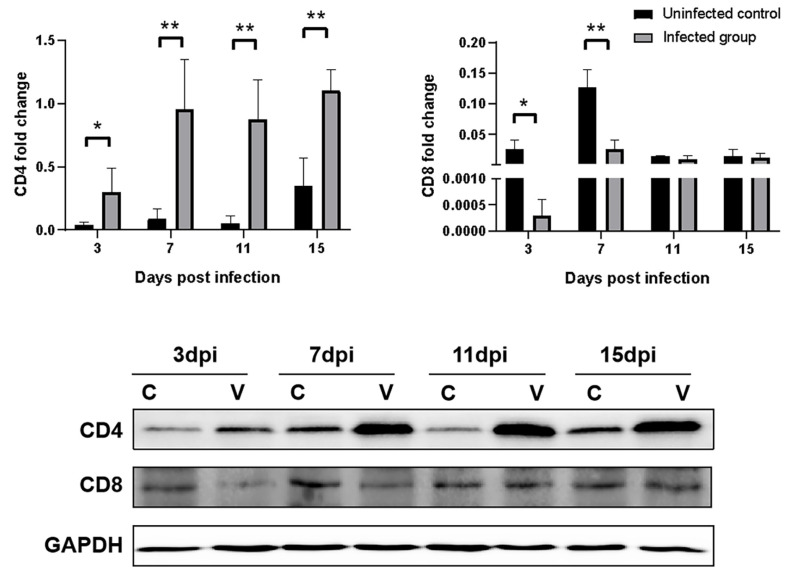
Changes in the protein levels of CD4 and CD8 in the spleen determined by Western blot at 3, 7, 11, and 15 dpi after infection with GNAstV. C: uninfected control group; V: GNAstV-infected group. Values are expressed as mean ± SD, *n* = 5. * *p* < 0.05; ** *p* < 0.01.

The expression of genes and proteins associated with antigen presentation molecules (CD4 and CD8) in the spleens was determined by IHC and Western blot. Both the control group and the infected group had positive signals for CD4 expression (Figure 9A), and statistical analysis showed that CD4 was significantly increased in the infected group at 3, 7, 11, and 15 dpi compared with the negative control group (Figure 9B; *p* < 0.05), while CD8 was significantly decreased in the infected group at 3 and 7 dpi compared with the control group (Figure 9C, *p* < 0.5). Similar results were obtained for CD4 and CD8 protein expression (Figure 10), and the CD4 protein levels were higher in the infected group at 3, 7, 11, and 15 dpi than that of the control group (*p* < 0.5), while expression of the CD8 protein was lower in the infected group at 3 and 7 dpi than that of the control (*p* < 0.5).

## 4. Discussion

The spleen is an important immune organ, and plays a vital role in host immune responses against invading pathogens in geese. In the study, GNAstV infection was shown to cause lymphocyte depletion in the spleen, especially during early stages of infection, indicating that GNAstV induces immune suppression in goslings. The depletion of lymphocytes was alleviated in later stages of GNAstV infection, which could be related to improvement of host immune function, as the goslings grew older. Young goslings are more easily infected with GNAstV than older goslings, and goslings at 25 and 35 days of age only exhibit mild symptoms following GNAstV infection [20]. In addition, lymphocyte depletion was mainly observed around the white pulp of the spleen in GNAstV-infected goslings, which may be associated with the blood-spleen barrier (BSB) of the spleen. The BSB is a filter bed for red blood cells that protects against microorganisms, and is located in the ellipsoid and periellipsoidal lymphatic sheath (PELS) in avians, which are composed of the white pulp [21]. These data suggest that GNAstV in the blood may accumulate in the BSB, and subsequently cause damage to lymphocytes around the BSB. It has been reported that duck Tembusu virus particles are primarily distributed in the PELS within the spleen’s white pulp, and can cause vacuolar degeneration in the PELS [22]. Reticular cells are stromal cells that play an important role in the migration and positioning of lymphoid cells [23]. The pathological changes of reticular cells observed in this study indicate that GNAstV infection may damage lymphocyte development and immune function. Moreover, reticular fibers are important components of the supporting framework in the spleen that provide channels and specific microenvironments for the migration of T and B lymphocytes [24,25]. In the study, GNAstV infection caused reduction and rupture of reticular fibers, which may also contribute to spleen damage.

Apoptosis is important in the defense against viral infection. HosT-cells quickly initiate apoptotic processes following stimulation by viruses, and restrict viral replication by clearing the infected cells [26]. In this study, GNAstV infection induced lymphocyte apoptosis in the spleen, suggesting that apoptosis in splenic lymphocytes may be a primary mechanism to combat GNAstV infection in goslings. Apoptosis can be triggered by diverse cellular signals, such as the death receptor-mediated extrinsic pathway, the intrinsic mitochondrial pathway, and the endoplasmic reticulum stress-mediated pathway. The Fas/FasL signaling pathway is one of the major death receptor-mediated extrinsic apoptosis pathways. In this study, GNAstV infection induced the mRNA up-regulation of Fas and FasL, indicating that the death receptor-mediated extrinsic apoptosis pathway is activated following GNAstV infection. GNAstV was found in lymphocytes and macrophages in this study, and lymphocyte apoptosis may be directly caused by GNAstV. However, indirect effects that induce apoptosis should not be excluded. The mechanism of apoptosis in splenic lymphocytes induced by GNAstV needs to be further investigated.

CD4 T-cells can recognize the MHC-II antigen, and CD8 T-cells can recognize the MHC-I antigen. The interaction between CD4 and CD8 T-cells and MHC molecules can enhance the binding of MHC molecules with T-cell receptors, which is important for immune defenses in the avian species. In this study, GNAstV infection increased levels of CD4 and decreased levels of CD8 at the early stages of infection. CD8 plays an important role in clearing cells infected with a virus. The reduction of CD8 induced by GNAstV may indicate a reduction in the host immunity of goslings, which would facilitate virus proliferation at the beginning of infection. The decrease of CD8 numbers may be related to the apoptosis observed in splenic lymphocytes, although this would need to be confirmed in future studies. In addition, we previously reported that the mRNA expression level of CD8 T-cells is increased in spleens of goslings infected with GNAstV [11]; the mRNA expression level of CD8 T-cells was also increased in the present study (data not shown). It is interesting that the mRNA results are opposite to the protein results; this may be related to the post-transcriptional mechanism, such as protein translation and post-translational modification and degradation, but the detailed mechanisms need to be further investigated.

In conclusion, GNAstV localizes to splenic lymphocytes and macrophages in the spleens in infected goslings. In the spleen, GNAstV infection causes lymphocyte depletion, reticular cell necrosis, reticular fiber destruction, lymphocyte apoptosis, and CD8 T-cell reduction. These pathological changes result in significant injury to the spleen. Our results also suggest that GNAstV can induce immunosuppression, which may facilitate infection by other pathogens.

## Figures and Tables

**Table 1:** Primers used in the study for real-time PCR.

Primers	Accession Number	Primer Sequences (5′-3′)	Host	Product Length (bp)
Beclin1-F	XM_013199763.1	CGCTGTGCCAGATGTGGAAGG	Anser	151 bp
Beclin1-R		CAGAAGGAATACTGCGAGTTCAAGA [18].		
ATG5-F	XM_013175657.1	CCGATTGGTTTGCTCTT	Anser	226 bp
ATG5-R		ATCCCATCCACAGTTGC		
Bax-F	KY788660	GGACGAGCTGGACAGCAACG	Anas	159 bp
Bax-R		AGGCGGCAGGCGAAGTAGA		
Bcl-2-F	XM_013187395.1	TGACCGAGTACCTGAACCG	Anser	154 bp
Bcl-2-R		GCTCCCACCAGAACCAAA		
cyt C-F	XM_013171877.1	AAATGCTCCCAGTGCC	Anser	159 bp
cyt C-R		CATCAGAGTATCCTCACCC		
Fas-F	XM_013171650.1	CACTCCCACAAGTCAAG	Anser	163 bp
Fas-R		AGTAGGGTTCCATAGGC		
FasL-F	XM_01319016.1	ATGGAAGATCACGCAAAGC	Anser	152 bp
FasL-R		GGTGGGAAGGGAGCAAT		

## Data Availability

The data that support the findings of this study are available from the corresponding author upon reasonable request.

## References

[1] Zhang Q., Cao Y., Wang J., Fu G., Sun M., Zhang L., Meng L., Cui G., Huang Y., Hu X. (2018). Isolation and characterization of an astrovirus causing fatal visceral gout in domestic goslings. Emerg. Microbes. Infect..

[2] Chen H., Zhang B., Yan M., Diao Y., Tang Y. (2020). First report of a novel goose astrovirus outbreak in Cherry Valley ducklings in China. Transbound. Emerg. Dis..

[3] Wei F., Yang J., Wang Y., Chen H., Diao Y., Tang Y. (2020). Isolation and characterization of an astrovirus causing fatal visceral gout in domestic goslings. Emerg. Microbes. Infec..

[4] Li J., Hu W., Liu T., Zhang H., Opriessnig T., Xiao C. (2021). Isolation and evolutionary analyses of gout-associated goose astrovirus causing disease in experimentally infected chickens. Poult. Sci..

[5] He D., Yang J., Jiang X., Lin Y., Chen H., Tang Y., Diao Y. (2020). A quantitative loop-mediated isothermal amplification assay for detecting a novel goose astrovirus. Poult. Sci..

[6] Chen Q., Xu X., Yu Z., Sui C., Zuo K., Zhi G., Ji J., Yao L., Kan Y., Bi Y. (2020). Characterization and genomic analysis of emerging astroviruses causing fatal gout in goslings. Transbound. Emerg. Dis..

[7] Yin D., Yang J., Tian J., He D., Tang Y., Diao Y. (2020). Establishment and application of a TaqMan-based one-step real-time RT-PCR for the detection of novel goose-origin astrovirus. J. Virol. Methods.

[8] Zhang X., Ren D., Li T., Zhou H., Liu X., Wang X., Lu H., Gao W., Wang Y., Zou X. (2018). An emerging novel goose astrovirus associated with gosling gout disease. Emerg. Microbes. Infec..

[9] Niu X., Tian J., Yang J., Jiang X., Wang H., Chen H., Yi T., Diao Y. (2018). Novel Goose Astrovirus Associated Gout in Gosling. Vet. Microbiol..

[10] Wang Z., Li L., Liu P., Wang C., Lu Q., Liu L., Yang Y., Luo Q., Shao H. (2021). Host innate immune responses of geese infected with goose origin nephrotic astrovirus. Microb. Pathog..

[11] Wu W., Qiu S., Huang H., Xu R., Bao E., Lv Y. (2021). Immune-related gene expression in the kidneys and spleens of goslings infected with goose nephritic astrovirus. Poult. Sci..

[12] Liu H., Hu D., Zhu Y., Xiong H., Lv X., Wei C., Liu M., Yin D., He C., Qi K. (2020). Coinfection of parvovirus and astrovirus in gout-affected goslings. Transbound. Emerg. Dis..

[13] Wu W., Xu R., Lv Y., Bao E. (2020). Goose astrovirus infection affects uric acid production and excretion in goslings. Poult. Sci..

[14] Fischer A.H., Jacobson K.A., Rose J., Zeller R. (2008). Hematoxylin and Eosin Staining of Tissue and Cell Sections. Cold Spring Harb. Protoc..

[15] McNeilly F., Allan G.M., Moffett D.A., McNulty M.S. (1991). Detection of chicken anaemia agent in chickens by immunofluorescence and immunoperoxidase staining. Avian Pathol..

[16] Qiu S., Xu R., Guo Y., Lv Y. (2020). Establishment of a SYBR Green I-based real-time PCR for goose nephritic astrovirus. Chin. Vet. Sci..

[17] Sun J., Zhang X., Cao Y., Zhao Q., Bao E., Lv Y. (2016). Ovarian Toxicity in Female Rats after Oral Administration of Melamine or Melamine and Cyanuric Acid. PLoS ONE.

[18] Lou Y. (2015). Study on Follicular Granulisa Cell Autophay Induced by Oxidative Stress in Goose. Master’s Thesis.

[19] Huang B., Li J., Zhang X., Zhao Q., Lu M., Lv Y. (2017). RIG-1 and MDA-5 signaling pathways contribute to IFN-β production and viral replication in porcine circovirus virus type 2-infected PK-15 cells in vitro. Vet. Microbiol..

[20] An D., Zhang J., Yang J., Tang Y., Diao Y. (2020). Novel goose-origin astrovirus infection in geese: The effect of age at infection. Poult. Sci..

[21] Xu M., Li W., Yang S., Sun X., Tarique I., Yang P., Chen Q. (2020). Morphological characterization of postembryonic development of blood–spleen barrier in duck. Poult. Sci..

[22] Sun X., Li W., Liu E., Huang H., Wang T., Wang X., Shi Y., Yang P., Chen Q. (2019). In vivo cellular and molecular study on duck spleen infected by duck Tembusu virus. Vet. Microbiol..

[23] Perez Shibayama C., Gil Cruz C., Ludewig B. (2019). Fibroblastic reticular cells at the nexus of innate and adaptive immune responses. Immunol. Rev..

[24] Pellas T.C., Weiss L. (1990). Migration pathways of recirculating murine B cells and CD4+ and CD8+ T lymphocytes. Am. J. Anat..

[25] Fukuta K., Mochizuki K. (1982). Formation of reticular fibers in the developing spleen of the chick embryo. Arch. Histol. Jpn. (Nihon Soshikigaku Kiroku).

[26] Carruthers V.B., Cotter P.A., Kumamoto C.A. (2007). Microbial Pathogenesis: Mechanisms of Infectious Disease. Cell Host Microbe.

